# On the effect of low oxygen concentrations on bacterial degradation of sinking particles

**DOI:** 10.1038/s41598-017-16903-3

**Published:** 2017-12-01

**Authors:** Frédéric A. C. Le Moigne, Carolina Cisternas-Novoa, Judith Piontek, Marie Maßmig, Anja Engel

**Affiliations:** GEOMAR, Helmholtz Centre for Ocean Research Kiel, Düsternbrooker Weg 20, D-24105 Kiel, Germany

## Abstract

In marine oxygen (O_2_) minimum zones (OMZs), the transfer of particulate organic carbon (POC) to depth via the biological carbon pump might be enhanced as a result of slower remineralisation under lower dissolved O_2_ concentrations (DO). In parallel, nitrogen (N) loss to the atmosphere through microbial processes, such as denitrification and anammox, is directly linked to particulate nitrogen (PN) export. However it is unclear (1) whether DO is the only factor that potentially enhances POC transfer in OMZs, and (2) if particle fluxes are sufficient to support observed N loss rates. We performed a degradation experiment on sinking particles collected from the Baltic Sea, where anoxic zones are observed. Sinking material was harvested using surface-tethered sediment traps and subsequently incubated in darkness at different DO levels, including severe suboxia (<0.5 mg l^−1^ DO). Our results show that DO plays a role in regulating POC and PN degradation rates. POC(PN) degradation was reduced by approximately 100% from the high to low DO to the lowest DO. The amount of NH_4_^+^ produced from the pool of remineralising organic N matched estimations of NH_4_^+^ anammox requirements during our experiment. This anammox was likely fueled by DON degradation rather than PON degradation.

## Introduction

Concentrations of dissolved oxygen (DO) may decline in the future ocean, potentially leading to an expansion of oxygen minimum zones (OMZs)^1,2^. Although earth system models do not all project increased hypoxia^3,4^ and although the causes of this expansion are still debated^5^, over the past 50 years supporting evidence for this occurring has been gathered globally^6^. In well-oxygenated waters, temperature^7,8^ and plankton community structure^9–12^ are known as the main factors affecting the particulate organic carbon (POC) degradation rate. In oxygen deficient waters however, the importance of temperature and plankton community on remineralisation rate may differ as oxygen itself may play a role^13–17^. Several studies^13,15–17^ in OMZs have based their estimates of POC degradation rate only on the attenuation of particle flux^18^. Only refs^14,17^ used direct estimates of particle degradation by looking at dissolved CO_2_ variations in enclosed degradation experiments. Also, the quality of the organic matter may play a role in dictating the sensitivity of degradation as the remineralisation rate of N-rich amino acids seems to be less affected by DO deficiency than that of non amino acid POC components^15,19^.

In OMZs, enhanced POC fluxes could be explained by (1) the fact that microbial respiration using non-O_2_ electron acceptors may not be as energetically efficient as in oxygenated waters, leading to lower particles remineralisation rates, or by (2) the low abundance in zooplankton may lead to the loss of the packaging function. This would in turn reduce the amount of fast sinking particles^20^ produced from a pool of slow sinking (or suspended) particles present within the OMZ via grazing and production of fecal pellets.

It is therefore not clear whether previously observed trends in POC degradation rate in the studies cited above were only caused by the low DO concentrations or by differences in ecosystem structure and/or temperature and/or organic matter quality. To mechanistically test the impact of DO concentrations on POC degradation rate, one would therefore need to examine the degradation of POC under experimental conditions where only the DO concentration is changed. This implies the need for a survey of two regions where oxygen varies but all other environmental conditions such as temperature, organic matter quality, and important aspects of community structure such as bacterial, phytoplankton and zooplankton composition are fixed. This is however challenging, given that sediment trap deployments in the ocean are technically difficult to perform and artificial aggregates often differ considerably from natural ones.

Remineralising particulate nitrogen (PN) fuels NH_4_^+^ production that subsequently sustains the production of N_2_ gas (via anammox). Whether anammox or denitrification is the dominant process in OMZ is still debated^21–23^. Sinking OM could thus be degraded through alternative pathways^17^. More precisely, the heterotrophic microbial community in OMZs preferentially uses N-rich OM, leading a strong decoupling between C and N. This preferential use of N-rich organic matter could be responsible of a large loss of N_2_ from the ocean. However, PN flux estimates (and subsequent NH_4_^+^ production from remineralization) may not be sufficient to fully sustain the observed loss of N_2_ induced by anammox and denitrification^24^. This suggests that either (1) another source of NH_4_^+^ production occurs in oxygen deficient zones, with microaerobic respiration proposed as a source of ammonium during organic matter degradation^24^, or (2) estimates of NH_4_^+^ production rate from remineralisation of PN presented in ref.^21^ could be biased low, either via underestimation of surface PN export/remineralisation rate or omission of NH_4_^+^ production from the DOM pool which is, on occasion, supplied below the oxycline^25^. Collectively, this highlights the high current uncertainty in the contribution of PN degradation to the N budget in OMZ.

The discussion above provides motivation for direct measurements of export and OM remineralisation in oxygen deficient areas. Here, we present results from a bottle degradation experiment testing the influence of DO concentration on POC and PN degradation. Sinking material was collected using surface tethered sediment traps in the Gotland Deep (Baltic Sea), where DO concentrations are close to detection limit at 100–150 m. Harvested sinking material was subsequently incubated for 6 days in seawater, replete with associated microbial communities, collected at the same depth as the sediment traps in order to test the effect of different DO concentrations on POM degradation. Particulate OC (POC) and N (PN) were monitored every other day alongside the geochemical quality of the organic matter (using a degradation index^26^) and the bacterial abundance and biomass. Nutrients concentrations were also measured and used as indicators of OM microbial regeneration pathways.

## Results and Discussion

### Biogeochemical conditions

**Figure 1:**
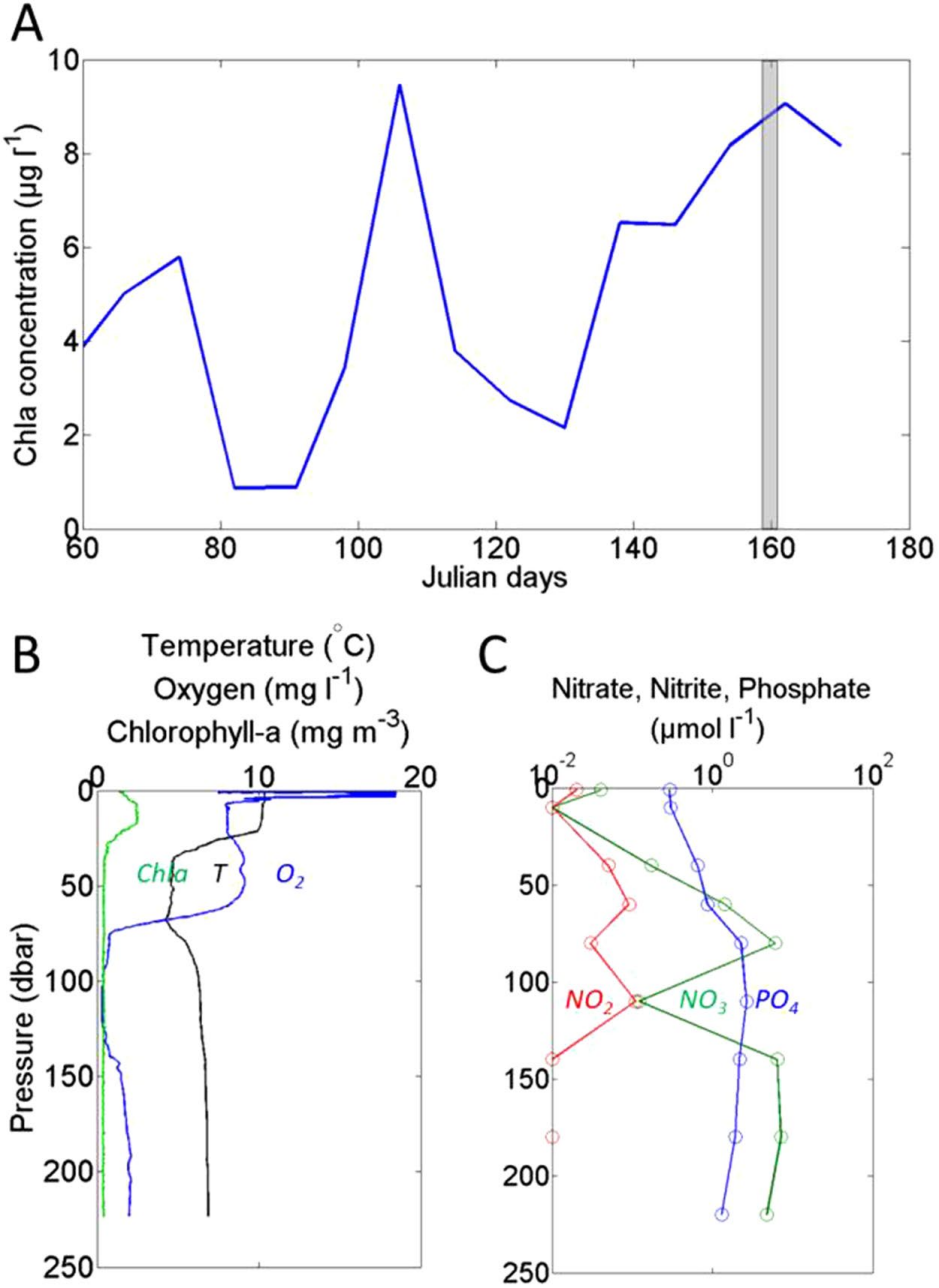
(**A**) Satellite chlorophyll a concentration time series (MODIS) centered around the sampling site (see methods). Days are in Julian days and the trap deployment period is highlighted in grey. Vertical profiles of (**B**) temperature, fluorescence-derived chlorophyll-a, and DO, (**C**) nitrite, nitrate and phosphate, at the sampling site. Note the log_10_ scale used in panel (**C**).

surface satellite-derived Chl-a concentrations in the Gotland Deep peaked in mid-June (8–10 mg m^−3^, Julian day 160) during sampling (Fig. 1A). Chl-a concentrations increased constantly from mid-May (Julian day 135) to the sampling period. Figure 1B shows vertical profiles of temperature (°C), Chl-a (mg m^−3^) and DO concentration (mg l^−1^). Surface temperature (10 °C) dropped to 5 °C between depths of 35 and 75 m. Temperature then increased to 8 °C below 75 m and remained stable down to 220 m. Chl-a peaked in the subsurface (2–3 mg m^−3^) and decreased to background levels below 35 m. DO concentrations decreased significantly below 60 m, reaching a minimum of 0.4 mg l^−1^ at 110 m, which is typical for the deep Baltic Sea^27^. However, below 140 m DO concentrations increased up to 3 mg l^−1^. This was likely due to an inflow of dense North Sea water that had previously entered the Baltic Sea during the strong winter storms observed in late 2014–early 2015^28,29^.

Phosphate (PO_4_^3−^) and nitrate (NO_3_^−^) concentrations increased with depth down to 75 m (2.3 and 6.1 µmol l^−1^ respectively). Below 75 m, phosphate remained constant, but NO_3_^−^ decreased strongly down to 0.1 µmol l^−1^ at the depth of the DO concentration minimum (110 m). Nitrite (NO_2_^−^) concentration peaked at the same depth, i.e. 110 m with 0.1 µmol O_2_ l^−1^. This suggests a preferential use of NO_3_^−^ relative to PO_4_^3−^. Plotting our estimated Baltic N* values^30^ (see methods) versus DO concentration (Figure S1), it clearly appears that the excess of PO_4_^3−^ is related to DO deficiency in the water column. Along with the nitrite accumulation observed at 110 m (Fig. 1C), this suggests that losses of N, likely through denitrification processes related to low DO concentrations, occurred in the water column.

In the following section, we describe the effect of DO concentration on the degradation of sinking POM. We collected sinking material using surface tethered sediments traps at various DO levels including oxic, suboxic and anoxic concentrations (Fig. 1B) corresponding to the following depths (40, 60, 110, 180 m)^31^. We then incubated (12 °C, complete darkness, 6 days) sinking material in *in situ* water (collected with CTD rosette) in gas-tight glass bottles, keeping the DO levels at similar concentrations as in the water column (at 40, 60, 110, 180 m depths, Fig. 1B). Particles were maintained in constant suspension using a modified plankton wheel rotating at 2 rpm (rotation per minute). We estimated how quantitative our sampling procedure was by looking at the variations in total Si (dissolved silicate + biogenic silica concentrations). Our attempt at completing a budget for Si failed due to uncertainties related to the sampling procedure, which ranged from 0 (day 4, 40 m) to 22% (day 4, 60 m). On average, uncertainties due to the sampling procedure was 4% in the 40 m samples, 12% in the 60 m samples, 8% in the 110 m samples, and 3% in samples collected at 180 m (Figure S2). These errors were estimated from the duplicates samples of both DSi and BSi (Figure S2).

### Organic matter dynamics during the incubation experiments

**Figure 2:**
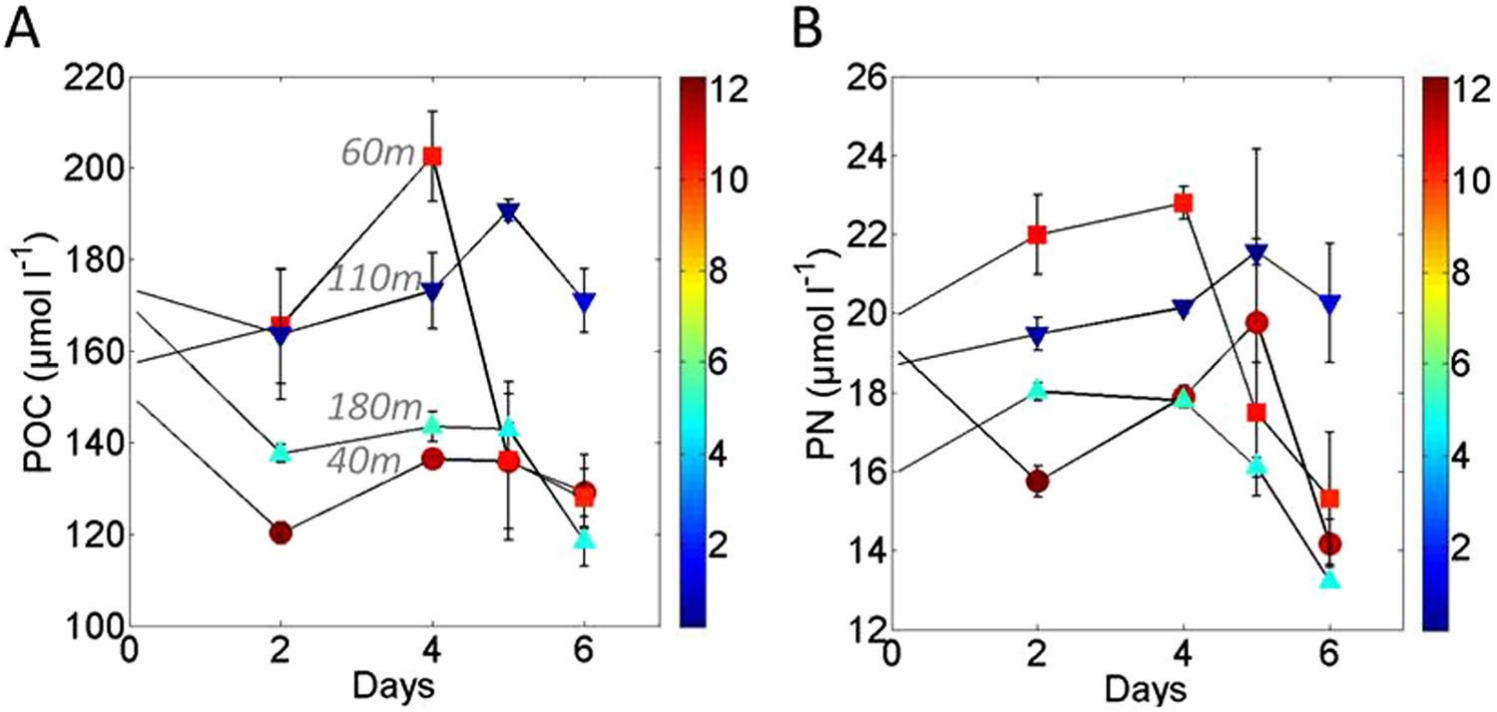
Variations in (**A**) POC and (**B**) PN concentrations (µmol l^−1^) over the course of the experiment. Error bars indicate the standard deviation from the duplicate samples. Circles represent 40 m samples, squares 60 m, inverted triangles 110 m and triangles 180 m samples. The DO concentration (mg l^−1^) in each bottle (when taken down) is indicated by the colour scale in both (**A**) and (**B**) panels.

Figures 2A and B present the variations of POC and PN concentration (µmol l^−1^) in the incubation bottles over the time course of the experiment. At the start of the experiment DO concentrations in experimental bottles corresponding to depths of 40, 60 110 and 180 m were 10.0, 11.4, 0.5, and 5.7 mg l^−1^ respectively. Although these are slightly different from the DO concentrations observed on site, they correspond to the depth pattern measured in the water column within the vicinity of the trap sampling site^31^ (Fig. 1B). The concentration of DO measured in individual artificial aggregates has previously been found to decrease from the periphery to the core of artificially produced aggregates^32^. Suboxia has been observed within the core of aggregates when the concentration of DO in the background water fell below 1.9 mg l^−1^ ^33^. We thus assume anoxia within the sinking particles collected at 110 m in our experiment. However, sinking particles collected in the 180 m treatment may still have had sufficient DO concentrations for microbes to perform aerobic respiration (Fig. 1B) as previous studies^34^ have showed that microbial activity switches from oxic respiration to N-based respirator pathways at concentrations less 0.1 mg l^−1^.

At day 0, POC and PN concentrations varied between the different depth treatments (POC: 148 µmol l^−1^ for 40 m to 172 µmol l^−1^ for 110 m, Fig. 2A; PN: 16 µmol l^−1^ for 180 m to 20 µmol l^−1^ for 60 m, Fig. 2B). However, POC(PN) concentrations varied widely during the first days of the experiment, including increases in POC(PN) (Fig. 2A). Such increases in enclosed systems (the so-called ‘bottle effect’) is on occasion observed in the initial days of such experiments before stabilization^11,12^. This may be associated with constant exchanges of the organic carbon (OC) between the dissolved and the particulate phase and/or bacterial growth^12^.

In order to assess the variability in the concentration of POC(PN) with time during our experiment, we performed repeat linear regressions (POC, PN, DOC concentrations vs time) that included and excluded low DO samples (110 m). The results from the regressions are summarized in Table S3. We found that the concentration of DOC was significantly correlated (p < 0.05) with time (Table S3) at all depths and also when excluding 110 m samples. Their respective slopes (Table S3) were however not significantly different. When considering all time steps, no significant relationships were found between both POC:PN ratios, POC(PN) concentrations and time (Table S3). We also tested our results using data from days 4–6 only, based on the patterns observed in Fig. 2A and B. Using these time steps no correlations were found between POC:PN ratios or DOC concentration and time. Likewise, no correlations were found between POC(PN) concentrations and time. However, when 110 m samples were excluded from the regressions, POC(PN) concentrations showed significant decreases with time (Table S3).

**Figure 3:**
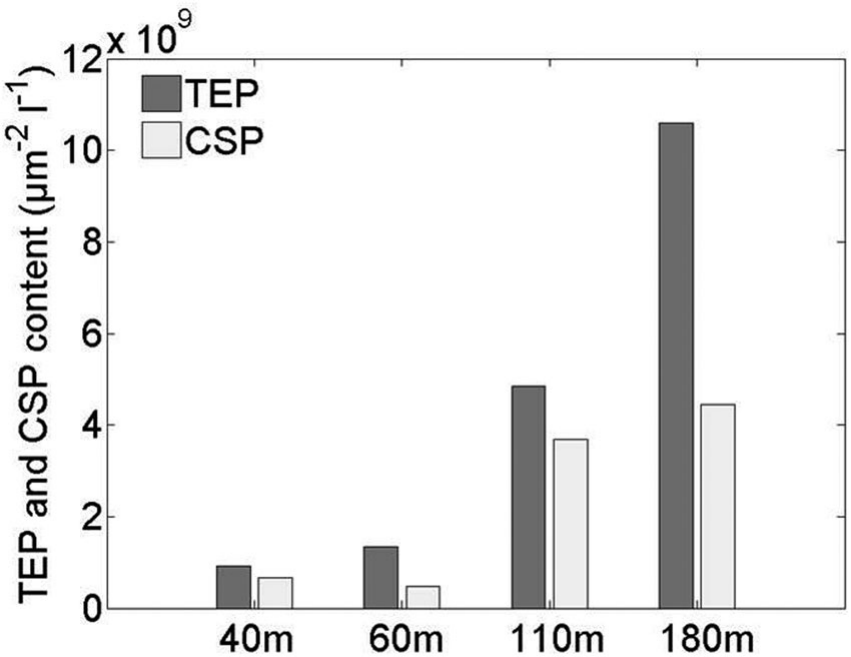
Content of transparent expolymers (TEP) and Coomassie Stainable Particles (CSP) at day 6 in µm^−2^ l^−1^ in the different depth treatments.

Processes of POM aggregation/dissolution and DOM adsorption/desorption are generally considered as being a function of ecological factors, such as mineral content of the sinking particles, but microorganisms can also participate in DOM/POM exchanges via alteration of the chemical nature of OM^35^ and solubilizing POM into DOM through cell lysis and release of ectoenzymes. Whilst we can exclude particle disaggregation by zooplankton, which we purposely eliminated here (see methods), disaggregation induced by the degradation of sticky organic matter by microbes cannot be excluded^36^. Abiotic factors such as sunlight can be ruled out in our experiment. Also the mineral content, such as biogenic silica, in the sinking particles was relatively low during at the study site^31^ in comparison to fluxes in other ocean provinces^37,38^, so we assumed little interaction of this component. The content of transparent exopolymere particles (TEP)^39^ and coomassie stainable particles (CSP)^40^ (Fig. 3) could be used to qualitatively estimate the transfer OM from the dissolved to particulate phase and could potentially explain the large augmentation in POM vs DOM during the initial part (days 0 to 4) of the experiment (note that the augmentation observed at 60 m day 4 is likely due to a quantitative sampling issue, as shown in Figure S2). We believe that DOM coagulation played an important role during our experiment. POM concentration increases (Fig. 2) were often accompanied by increases in C:N ratios. This supports the idea that DOM (C:N = 16.3 ± 1.6 on average) was adding to the POM pool (C:N = 8.2 ± 0.5 on average).

**Figure 4:**
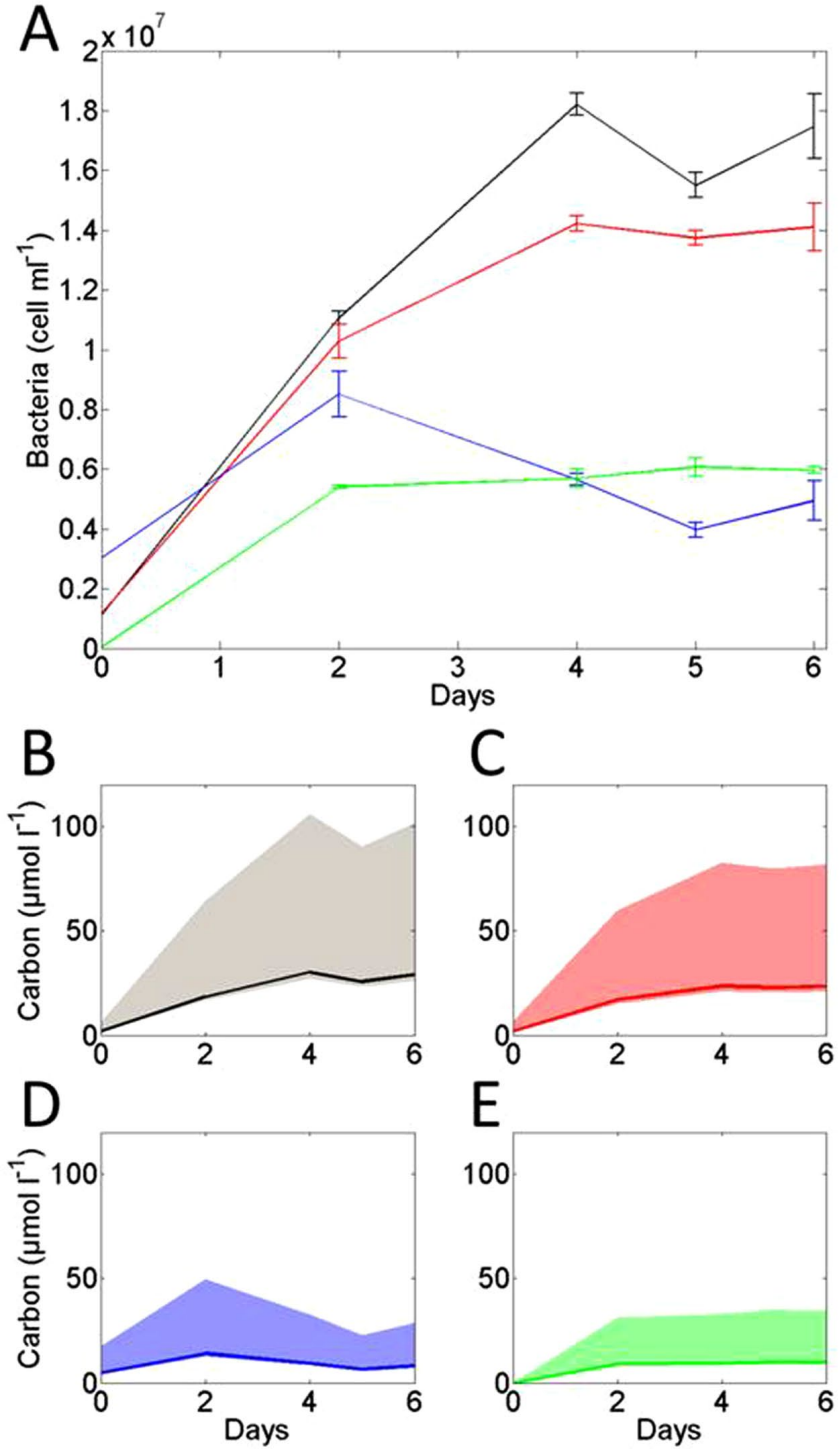
(**A**) Abundance of bacteria (cell ml^−1^) and associated standard deviation from duplicates. Black line represents 40 m samples; red, 60 m; blue, 110 m and green, 180 m. The estimated equivalent in carbon (µmol l^−1^) is presented in (**B**) for 40 m, (**C**) for 60 m, (**D**) for 110 m and (**E**) for 180 m. The bold line represents estimates following cell biomass conversion factor^42^, the shaded area around the line represents for lower and upper values using lower and values for the cell biomass conversion factor as described in ref.^41^. See text for more detailed information.

Bacterial growth is another factor that likely influenced POM concentrations during the experiment. Using cell counts, we estimated the amount of C resulting from bacterial growth using cell to carbon conversion factors^41,42^. Figure 4A shows the abundance of bacteria in the various samples during the experiment. The abundance of bacteria increased in all treatments, plateauing at day 2 in 110 m and 180 m treatments and day 4 in 40 m and 60 m treatments. We observed bacterial growth during our experiment, suggesting that particle associated bacteria recovered from exposure to the brine during collection. In the incubation bottles the salinity was close to *in situ* conditions given that particles were diluted in seawater collected at the same depths as the particles (see methods). The largest increase in bacterial abundance was observed in samples from 40 m, while the smallest increase was observed in 110 m samples. Bacterial abundance in samples from 60 m closely followed the pattern observed in 40 m samples, whereas abundance in samples from 180 m showed similar variations as the 110 m samples (Fig. 4A). We chose a conversion factor of 20 fgC cell^−1^ ^42^ to estimate the amount of carbon added to what we considered as POC (larger than 0.7 µm, see methods). The upper(lower) ranges using a conversion factor of 50(2) fgC cell^−1^ ^41^ are presented in Fig. 4B,C,D,E. Increases in organic carbon (OC) generated by bacterial growth from day 2 to day 6 (using 20 fgC cell^−1^) were 11, 6, −5 and 1 µmol l^−1^ for sample taken at 40, 60, 110 and 180 m respectively (Fig. 4B,C,D,E). Using the lower cellular carbon conversion factor (2 fgC cell^−1^) lowers these numbers 10 fold (Fig. 4B,C,D,E). Using the upper conversion factor (50 fgC cell^−1^), generates increases of 27, 15, -12 and 2 µmol OC l^−1^ for samples taken at 40, 60, 110 and 180 m respectively. Given our observed increase in POC of about 10 µmol l^−1^ in the 40 m samples, the conversion factor of 20 fgC cell^−1^ is appears the most applicable to use. Between day 4 and day 6 bacteria abundance was constant and hence contributed to no OC generation (Fig. 4A). The growing and changing microbial community (Fig. 4A) from day 0 to day 4 could have produced a significant amount of POC, as explained in the paragraph above (Fig. 4B–E). We however show that microbial community growth (and therefore potential creation of POC) was reduced after day 2, since the bacteria abundance was steady during three measurement time steps (day 2, 4, 5 and 6).

We therefore use degradation calculated between days 2 and 6. The bacterial community appeared to have reached a steady state condition from day 4 onwards, but DOM coagulation seemed to have occurred throughout the entire duration of our experiment. For both these reasons we chose to restrict our examination of POM degradation by looking at data from day 2, day 4, day 5 and day 6, and ignoring data from day 0. We do acknowledge that by adopting this approach we lose characterization of the initial step of degradation; however this is not of great concern for this study where the focus is on testing the mechanistic impact of DO concentrations on POM degradation rather the absolute consumption rates.

### POM degradation

**Figure 5:**
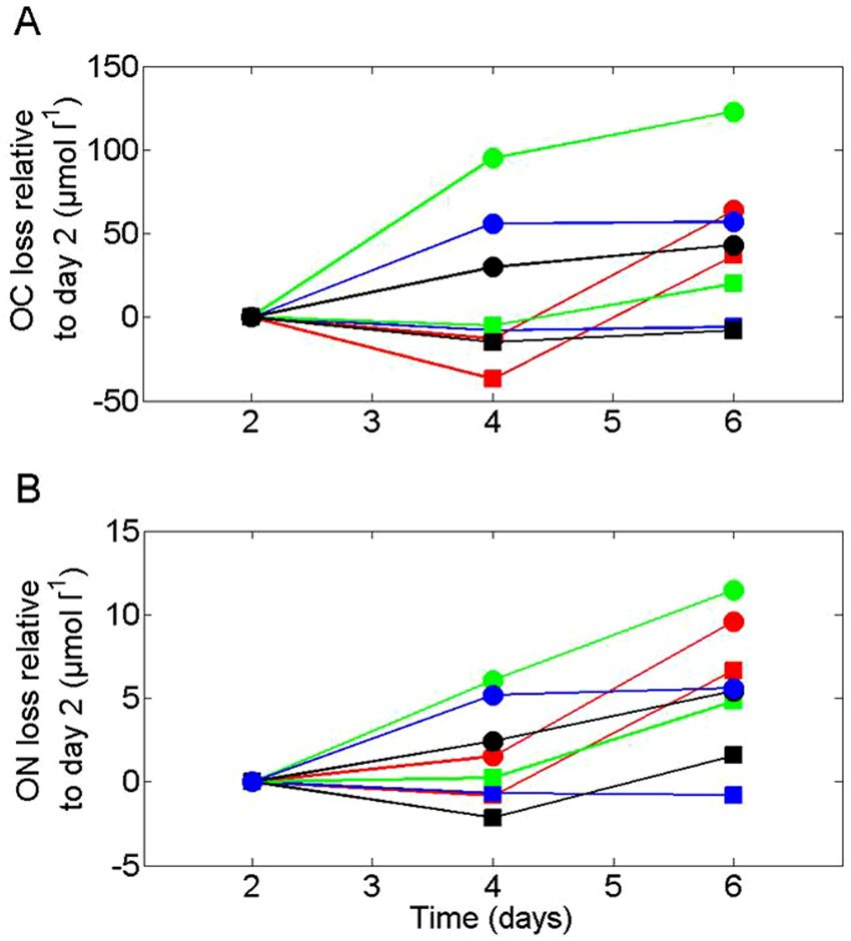
Losses of OC (**A**) and ON (**B**) in µmol l^−1^ as calculated from the difference in OC(N) concentrations relative to day 2. Circles represent TOC(N) and squares POC(N). Black lines/symbols represent 40 m samples; red, 60 m; blue, 110 m and green, 180 m.

We distinguished differences between the treatments by plotting losses of POC (Fig. 5A, Table S1) and PN (Fig. 5B, Table S2) in µmol l^−1^ as calculated from the difference in POC(PN) concentrations between days 2 and 6 (see justification above). Additionally, we calculated the losses of TOC and TN also as determined from the difference in TOC(N) concentrations during the experiment (Fig. 5).

POC loss (Fig. 5A) was the largest in 60 m depth samples (−37 µmol l^−1^). Surprisingly, ΔPOC_2–6_ was positive in the 40 and 110 m depth samples (9 and 7 µmol l^−1^ respectively). Our quantitative Si budget (Figure S2) suggested that the bottles corresponding to day 4 at 60 m had the largest deviation of all, suggesting that POC concentrations in this bottle could have been overestimated. We therefore believe that the large ΔPOC_2–6_ observed at 60 m should be treated as a potential overestimate. PN loss rates seem to follow the same pattern as POC (Fig. 5); again ΔPN at 110 m were negative. It is unclear why ΔPOC(N)_2–6_ at 40 and 110 m was negative (Fig. 2C and D). Rates of POC degradation were reduced by 116% from the high DO concentration to the low DO concentration (Table S1). The reduction is higher than 100% because of the small gain of POC observed in the low DO treatment with material from 110 m depth. Similarly, the reduction in PN degradation rates was about 100% (Table S2). Looking at the variation in concentrations of TOC (total organic carbon calculated by adding POC and DOC concentrations) and TON (total organic nitrogen calculated by adding PN and DON concentrations) relative to day 2 (Fig. 5), little OC or ON is lost in the 110 m samples while the larger loss occurs in the 180 m samples. Samples from 60 m however present only a small decrease in TOC, but a larger decrease in TON (Fig. 5). Temporally, changes seem to be greater in OC relative to ON. For instance, the variations of TOC and POC loss at 60 m (Fig. 5A, red symbols) are compared to the variations of ON at the same depth (Fig. 5B, red symbols). The trends we observed in both OC and ON degradation provide two main messages: (1) the material collected at 110 m seems to experience lower degradation rates than the material collected at other depths, and (2) while samples from 40 and 60 m have dissimilar degradation rates, the OM from 180 m have experienced the most intense remineralisation over the time course of our experiment. In the following section we investigate what causes the patterns in OC and ON degradation described above.

### Control on POC(PN) degradation rate, DO vs organic matter quality

As stated in introduction, DO concentration can potentially have a large impact on particle remineralisation rate^13,14,16,17^ through either influencing prokaryotic metabolic pathways of organic matter degradation^43^ or by reducing the production of fast sinking particles generated through zooplankton fecal re-packaging^20^. Zooplankton were purposely excluded (see methods section) during our experiment for the sake of focusing on bacterial degradation only.

To fully test the influence of DO concentrations of sinking particle degradation rate (induced by prokaryotic organisms), one would have to produce a homogenous cohort of sinking particles with similar morphological/geochemichal characteristics and then estimate their degradation rate *in vitro*. This was done previously using sinking particles artifically produced from phytoplankton cultures, however this presents the potential risk of including prokaryotic communities that differ from natural communities found in the field. Specifically, culture-associated prokaryotic communities^11,12^ differ from natural communities^44,45^ and are often less active in degrading sinking particles^46,47^. This is the reason why we here used natural aggregates. However, an implication of this is that we need to consider other parameters, in addition to DO concentration, which could potentially drive patterns in OC/ON degradation rates. We here consider an additional parameter linked to the quality of the organic matter: the degradation index (DI) which is based on the amino acid content of particles^26^.

**Figure 6:**
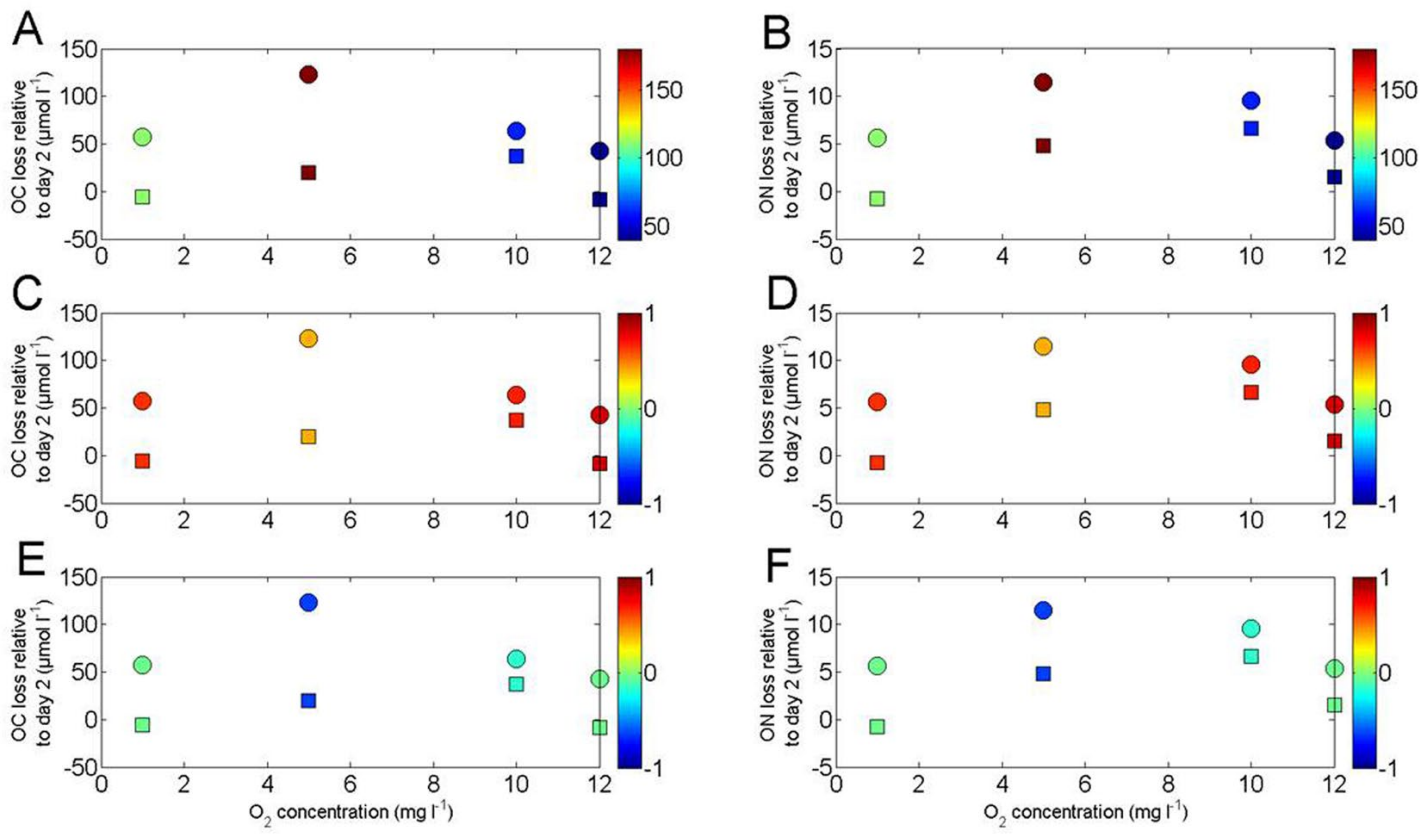
Losses of OC and ON in µmol l^−1^ (calculated from the difference in OC(N) concentrations at day 6 relative to day 2) as a function of DO concentration (mg l^−1^). Color bars represent (**A**,**B**) the sampling depth (m), (**C**,**D**) the degradation index^26^ of the material observed in sediment trap material before incubation and (**E**,**F**) the degradation index^26^ of the material observed in the incubation bottle at the end of the experiment (day 6). Circles represent TOC(N) and squares POC(N).

Figure 6 presents the losses of TOC(N) and POC(N) at day 6 relative to day 2 (from Fig. 5) as a function of DO concentration with depth (Fig. 6A,C) and the initial organic matter DI^26^ of trap material (start of incubation) and incubation bottle at day 6 on a color scale. We chose to look at losses at day 6 relative to day 2 as these cover the longest period of time, hence maximizing the signal to noise ratio. Although the reproducibility of such experiments is challenging to assess, we note that our TOC(N) and POC(N) losses provide independent support for these rates of 18(2)μM and 3(1) μM respectively. This is based on regressions between TOC(N) loss and sampling days. POC(N) losses co-vary with DO concentration if we only consider samples taken at 60, 110 and 180 m. If the 40 m samples are included, the relationship collapses. For TOC(N) losses we observed no trend relative to DO concentrations.

We find that in contrast to DOM, DO concentration is a potential driver for POM degradation. However, the differences in POC and PN losses observed between the samples taken from 40 and 60 m (with a similar DO concentration) indicate that factors other than DO concentration played a significant role in driving the magnitude of both POC and PN losses. It is unlikely that the reduced remineralisation rates observed in 40 m samples result from a lack/delay of colonization^48^ by bacteria, given the enhanced bacterial biomass at this depth (Fig. 4).

We hypothesize that the differences in degradation rates between 40 m and 60 m samples may be due to the OM composition^13^ or rates of chemoautotrophy. OM lability as diagnosed from the DI^26^ is a useful metric to compare OM composition. Lower DI indicates how much more degraded the OM is and *vice versa* ^26^. Figure 6C,D show the DI in the trap material before incubation (starting conditions) and Fig. 6E,F show the DI after the incubation (day 6). The DI is consistently higher in the surface relative to greater depths in both traps and incubation samples (Fig. 6). The DI was also consistently lower in the incubation compared to the traps at all depths. The DI in bottles from 40 m is higher (-0.05) than at 60 m (-0.18), consistent with the “starting point” observed in the trap material (0.82 at 40 m and 0.67 at 60 m). Given the similarity of the DIs (both in trap material and incubation bottles) at 40 and 60 m, but the large difference in POC loss rate between 40 and 60 m, we believe that DI has a moderate control on POC degradation rate at high DO concentrations. The influence of the lability of trap material on the POC degradation rates for samples from 110 (0.63) and 180 m (0.40) is less clear given their very different DO levels (Fig. 2) and cannot be tested. We did not measured depth resolved rates of chemoautotrophy at our experiment station, however chemoautotrophy rates were positively correlated with the concentration of NH_4_^+^ in June 2015 in the Baltic Sea^49^. At the location of the trap deployment the vertical profile of NH_4_^+^ concentration shows that concentrations at 110 m and 40/60 m were higher than at 180 m (Fig. S4). Assuming that chemoautotrophy rates follow the depth pattern of NH_4_^+^ concentrations^49^ this suggests that chemoautotrophy rates were similar at 40 and 60 m, and therefore cannot explain why POC and PN losses observed at 40 m are lower than the ones observed at 60 m. We propose that one possibility could be the amount of ballast mineral (biogenic silica, terrigenous material and calcite) present at 40 and 60 m, which is known to slow down POM remineralization^11,12,50,51^. Unfortunately the total mass flux was not measured, which prevents any conclusions being drawn with regards to the amount of mineral ballast present at each depth.

In any case, our results show that: (1) DO concentration restricts the POC(PN) degradation rate at low DO, and (2) POM degradation rates exhibited high variability at high DO, and may have been controlled by variability in degradation state, protection by ballast mineral, the nutrient environment, amongst other factors.

### Microbial remineralisation pathways

In the previous section we looked at the influence of DO concentration and OM composition on the rate at which particulate matter degraded. We now take a closer look at what may happen to the degraded particulate matter, specifically with regards to N. Although we do not have information on N transformation rates such as those provided by isotopic N compound labeling analyses^21,52,53^, we can investigate potential microbial metabolic pathways of remineralisation by looking at indirect evidence for N transformation rates, such as the variations in nutrients over time. We consider here the concentrations of NO_3_^−^, NO_2_^−^ and NH_4_^+^ following the N cycle under low DO conditions proposed in ref.^43^. Due to the complexity of the N cycle in low DO waters (see Fig. 3 in ref.^43^), the variations in nutrient concentration arises from five N remineralisation pathways: (1) ammonification (remineralisation of ON into NH_4_^+^), (2) anammox (conversion of NH_4_^+^ and NO_2_^−^ into N_2_), (3) denitrification (reduction of NO_3_^−^ and ON in N_2_O/N_2_), (4) nitrate reduction and (5) dissimilatory nitrate reduction to NH_4_^+^ (DNRA, reduction of NO_3_^−^ and ON into NH_4_^+^) during our experiment.

**Figure 7:**
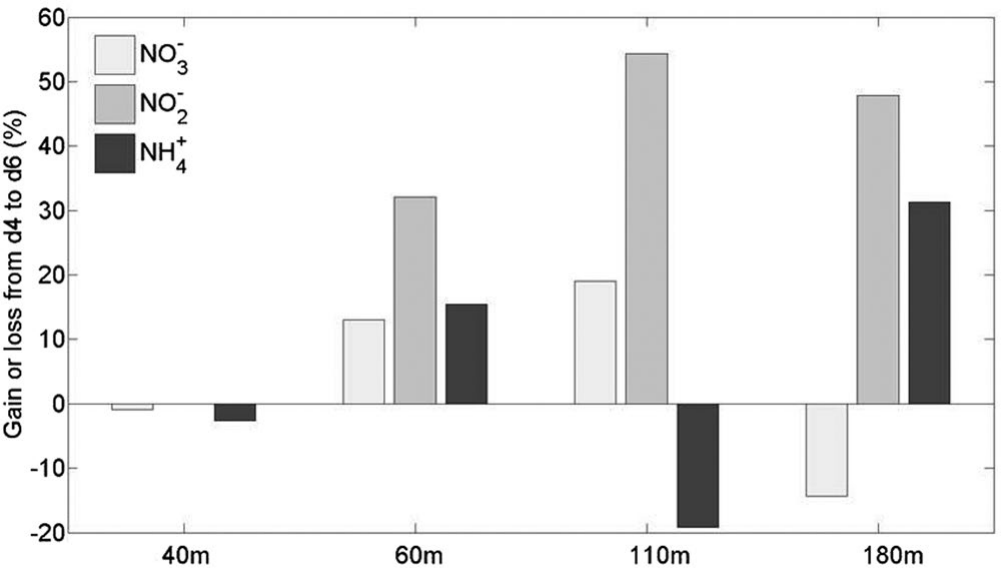
Variations of NO_3_^−^, NO_2_, NH_4_^+^ (as expressed as percentages of gain or loss between day 4 and day 6) over the course of our experiment. Negative percentages represent a decrease in concentration (consumption) from day 4 to day 6 while positive percentages represent an increase over the same time scale (accumulation). The variations of nutrients concentration (µmol l^−1^) are presented in Figure S4.

We observed some loss in dissolved inorganic N (DIN) during our experiment (Table S2) in the bottles corresponding to 110 and 180 m samples. In samples from 40 and 60 m the total concentration of DIN increased over the course of the experiment. To further investigate the origin of this loss we plotted Fig. 7 that shows the gain/loss of NO_3_^−^, NO_2_^−^ and NH_4_^+^ concentrations between day 4 and day 6 as a percentage of the concentration at day 6 relative to the concentration at day 4. Negative percentages represent a decrease in concentration (consumption/reduction) from day 4 to day 6, while positive percentages represent an increase over the same time scale (accumulation/production). The variation in nutrient concentration (µmol l^−1^) over the course of the experiment is presented in Figure S5. This approach was used to concisely and qualitatively illustrate nutrient variations during our experiment (Fig. 7). NO_3_^−^ increased or remained stable for all depth treatments apart from 180 m. It is unclear why NO_3_^−^ concentration decreased with time (Fig. 7) for the 180 m treatments. However, this has been observed in other degradation studies^11^ and is potentially due to the balance between the actual remineralisation of ON and the reduction of NO_3_^−^ through pathways 3, 4 and 5 (see text above). NO_2_^−^ concentration increased in all depths treatments; however, the increase observed in samples from 110 m was larger than at other depths while NO_2_^−^ concentration did not change in the 40 m samples. Similarly, NH_4_^+^ concentration increased (or remain similar) in all the oxygenated depth treatments (40, 60, 180 m) but in 110 m sample, the NH_4_^+^ concentration decreased.

Nonetheless, in the 110 m samples the lack of accumulation of NH_4_^+^ could result from less (or no) ammonification given the small amount of ON degraded between day 4 and day 6 (see previous section). This however does not explain the consumption of NH_4_^+^, which must result from anammox processes only. While our approach only provides indirect qualitative evidence, we speculate that anammox may have occurred in the Gotland basin. Anammox has previously been reported in the Gotland basin during some years^53^ while others^52^ found no evidence for it. One possible explanation is the stoichiometry of the OM. Loss of N through denitrification or anammox is primarily balanced by the stoichiometry of the OM. The lower the C:N ratio in sinking POM, the larger the proportion of annamox over total N loss^54^. It is then possible that late season N loss driven by denitrification observed in^52^ was due to different POM C:N ratios compared to those observed in spring (our study). In the Baltic Sea, sinking POM C:N ratios are known to show a large inter-annual variability^55^. This could potentially explain anammox in the Gotland basin in late spring.

It is possible to estimate anammox rates that could have occurred at 110 m in our samples. In the Baltic, anammox bacteria represent no more than 1.5% of the total abundance of bacteria^53^. In parallel, we know that in OMZs anammox bacteria have NH_4_^+^ consumption rates of 2–4 fmol cell^−1^ d^−1^ ^22,53,56^. This yields a consumption of 0.2 to 0.4 µmol l^−1^ d^−1^ of NH_4_^+^ by anammox bacteria (or 0.4–0.7 µmol l^−1^ of NH_4_^+^ consumed between day 4 and day 6). This is comparable with the total drawdown of NH_4_^+^ (0.2 µmol l^−1^) observed in 110 m samples between day 4 and day 6 (Figs 7 and S4). The consumption of ON in our 110 m bottles (Fig. 2B) between day 4 and day 6 was 0.4 µmol l^−1^ (Figure S3, Table S2). Using the relationship^21^ between PN export flux and annamox rates (anammox = 0.7 X PN export) would yield an NH_4_^+^ consumption of 0.3 µmol l^−1^. This matches our estimation of anammox rates described above. However, the loss of ON was mainly driven by the loss of DON and not PON (Figure S3, Table S2). We therefore believe that the consumption of NH_4_^+^ by anammox observed at 110 m may have been fueled primarily by ammonification from DON and not PN.

### Summary

Our results have relevance for two distinct processes. Firstly, for the drivers of the biological carbon pump, as it is currently unclear whether low DO concentration is the direct cause of reduced carbon flux attenuation observed in OMZs relative to oxygenated waters^14^ (and references therein). Our results suggest that:

1. DO concentration reduces the POC(PN) bacterial degradation rate at low DO concentrations, but other factors may be as important at higher concentrations. We found that the quality of the POM as determined from the DI index^26^ has a limited importance.
   Additionally, our experiment has relevance for the N cycle. The NH_4_^+^ produced from remineralising sinking PN is speculated to fuel anammox processes resulting in loss of N_2_ gas from the system^21^. However, it is unclear whether the NH_4_^+^ supply from remineralising sinking PN is sufficient to fully sustain the observed loss of N_2_ induced by anammox^24^. We show that:
2. NH_4_^+^ supply from remineralising organic nitrogen potentially matches estimates of anammox rates in the Gotland basin.
3. DON ammonification can also be a source of NH_4_^+^ for anammox bacteria.

## Methods

### Study Area, sampling and analysis of ancillary data

The June 2015 cruise took place onboard the F.S. Alkor (GEOMAR) in the Gotland basin (Fig. 1). Water samples were collected with Niskin bottles via deployment of a SeaBird CTD system. Dissolved oxygen concentration (DO) profiles were taken from the CTD optode and calibrated against DO concentration measurements performed using a semi-automated whole bottle Winkler titration unit^57^. Oxygen concentration in the incubations bottles was measured using a calibrated OXY-4 mini from Presens. In the bottles corresponding to 110 m samples (low DO concentration see Fig. 1), the DO concentration in the bottles at the start of the experiment was measured using Pyroscience sensor TROXR430 specifically designed for low DO concentration.

### Sediment traps deployment and sinking particles collection

Surface tethered sediment traps were deployed in the Gotland Deep on the 11^th^ of June and recovered on the 13^th^ (Fig. 1), Further details are provided in ref.^31^. The design of the traps and the drifting array follows^58^, with 12 individual Particle Interceptor Traps (PITs) mounted on a polyvinylchloride (PVC) cross frame^59^. Traps were deployed at four depths (40, 60, 110 and 180 m) with 12 PITs per depth. Prior to deployment, each PIT was filled with 1.5 l filtered surface seawater (0.2 µm pore size cartridge) collected from the ships underway seawater system, up to 3/4 of the PITs’s height. A brine solution was prepared by dissolving 50 g l^−1^ of NaCl with filtered surface seawater. This was subsequently filtered through a 0.2 µm cartridge to remove any particulate material. 0.5 l of this brine solution was then slowly pumped into each PIT with a peristaltic pump beneath the 1.5 l (3/4) of filtered seawater. Only the lowest 1/4 (0.5 l) were chosen to be filled with this solution to not lose the aspect ratio. Samples treatment we basically followed the recommendations by ref.^60^. Samples from each depth were flushed over a 500 µm mesh to remove zooplankton swimmers. Samples were subsequently split in aliquots five aliquots per depth, four were used for flux characterization^31^ and 1 for the experiment presented here. In the experiment aliquots, particles were left settling for four hours in the dark under controlled temperature (12 °C) in order to maximize the amount in particles transferred in the incubation bottles (see following section).

### Dissolved organic carbon, nitrogen and silica

DOC and TDN from CTD and experiment were analyzed from 20 ml samples filtered through 0.7 µm combusted GF/F filter onto pre combusted glass vials. Samples were analyzed using a TOC-VCSH (Shimadzu) analyser following procedures described in refs^61,62^. Nitrates (NO_3_^−^), nitrites (NO_2_^−^), phosphates (PO_4_^3-^), silicates (DSi) and ammonium (NH_4_^+^) concentrations from CTD and experiment were measured suing similar methods as in refs^63,64^.

### Particulate organic carbon, particulate nitrogen, biogenic silica, transparent exopolymers particle, coomassie stainable particles and amino-acids

Samples for POC and PN from CTD, traps and experiment were filtered onto a pre-combusted GF/F filters and stored at −20 °C until further analysis. Filters were then acidified over-night in an extractor in presence of concentrated fuming HCl. Filters were dried off and analyzed for C and N using a CHN analyser (Euro EA, Hechatech) following procedures described in ref.^65^. Samples for BSi were filtered at low vacuum (<200 mbar) onto 0.4 µm cellulose acetate filters and stored at −20 °C until further analysis. Filters were digested in NaOH at 85 °C for 135 mins, then the pH was raised to 8 using HCl and silicates were measured following ref.^66^.

Transparent exopolymere particles (TEP) and Coomassie stainable particles (CSP) from traps and experiment were analyzed by microscopy according to protocol presented in ref.^67^.

Dissolved free (DFAA), dissolved hydrolysable (DHAA), and total hydrolysable amino acids (THAA) were analyzed. For the dissolved amino acids, 20 mL of seawater were filtered through 0.45 μm syringe filters with low protein binding affinity (GHP membrane, Acrodisk, Pall Corporation). Total amino acids were analyzed directly in the unfiltered seawater sample. Samples were stored at −20 °C until analysis. For the determination of hydrolyzable amino acids, samples were hydrolyzed at 100 °C in 6 N HCl (Suprapur® Hydrochloric acid 30%) and 11 mM ascorbic acid for 20 h. Amino acids were separated by high performance liquid chromatography (HPLC), after ortho-phthaldialdehyde derivatization^68,69^, using a fluorescence detector (Excitation/Emission 330/445 nm). A pre-mixed standard containing: aspartic acid (Asp), glutamic acid (Glu), serine (Ser), arginine (Arg), glycine (Gly), threonine (Thre), alanine (Ala), tyrosine (Tyr), valine (Val), phenylalanine (Phe), isoleucine (Ileu), leucine (Leu), and γ-Aminobutyric acid (Gaba), was used for identification and quantification.

### Bacterial abundance

Bacterial cells from experiment were counted using a flow cytometer (FACSCalibur, Becton, Dickson). 4 ml of sample were collected, fixed with 200 µl of 25% glutaraldehyde and stored at -20 °C for further analysis. Samples were defrosted and sonicated to detach the particles-associated cells, filtered through a 50 µm mesh and analyzed on the flow cytometer following methods presented in ref.^70^.

### Incubation design

Sinking material from all depths (see section above) was then transferred into 1.2 l gas tight incubation bottles. 300 ml of sediment trap slurry split was mixed to 900 ml of CTD water sampled at the trap deployment location and depths. DO concentrations were then measured to control whether the concentration in the bottles actually corresponded to the actual DO concentration in the water column. The DO concentration in bottles corresponding to the DO minimum (110 m, see Fig. 2A) was significantly higher than the DO concentration observed at the same depth on the water column (Fig. 1A). Therefore, only the 110 m bottles were bubbled using a gas mix of N50 N_2_ and N45 CO_2_ (99.8675/0.1325%) to lower the DO concentration to the one observed in the water column.

Bottles were then place on a plankton wheel rotating at two rotations per minute (2 rpm) and kept in a temperature controlled room (12 °C) in complete darkness. Bottles were taken down on days 2, 4, 5 and 6 after the experiment. Clear aggregates were present in each bottle when taken down on sampling days. We measured DO, nutrients, ammonium, DOC, TDN concentrations, total bacteria abundance amino acid concentrations and particulate material POC, PN and TEP concentrations at every time steps.

## Electronic supplementary material

supplementary material
