# Supplementary material for: On the effect of low oxygen concentrations on bacterial degradation of sinking particles
Source: Sci Rep. 2017 Dec 1;7:16722. doi: 10.1038/s41598-017-16903-3 (PMC5711907; doi:10.1038/s41598-017-16903-3)
Supplement: Supplementary file 1 — supplementary material [file 41598_2017_16903_MOESM1_ESM.pdf]

<u>Supplementary material</u>

On the effect of low oxygen concentrations on bacterial degradation of sinking particles

Frédéric A.C. Le Moigne*, Carolina Cisternas-Novoa, Judith Piontek, Marie Maßmig, Anja Engel.

GEOMAR, Helmholtz Centre for Ocean Research Kiel, Düsternbrooker Weg 20, D-24105 Kiel

**Corresponding author:* Frédéric A.C. Le Moigne, GEOMAR, Helmholtz Centre for Ocean Research Kiel, Düsternbrooker Weg 20, D-24105 Kiel, Germany, +49 431 600-4141

flemoigne@geomar.de

Keywords: Export, Remineralisation, POM, Anoxia, Carbon, Nitrogen, denitrification, anammox

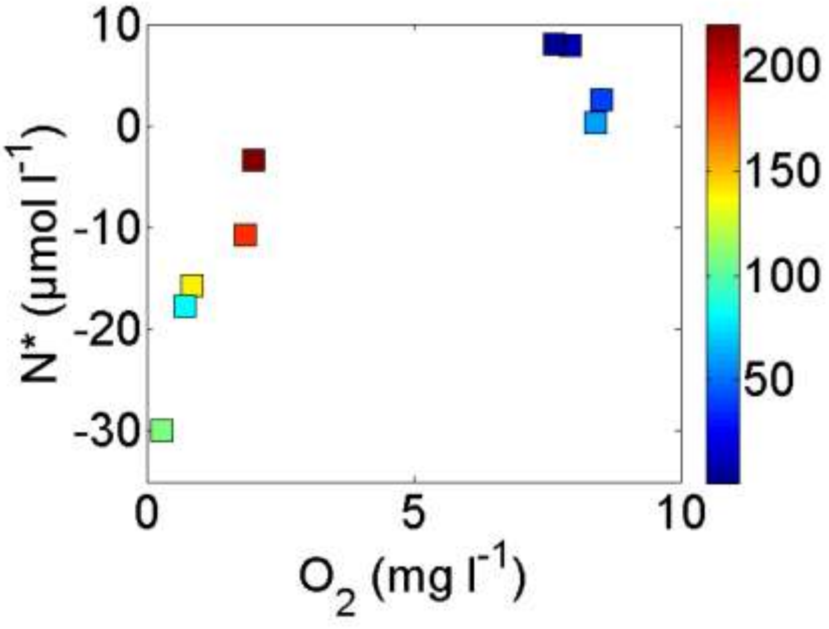

Figure S1: Water column N^*_{Baltic} (N^*_{Baltic} = nitrate – 16 x phosphate + 12.7 adapted from reference [65]; 12.6 was added to scale the June 2015 Baltic mean of N* to zero) versus O_2 concentration in the water column at the sampling site (see methods). Samples depth is indicated as color bar.

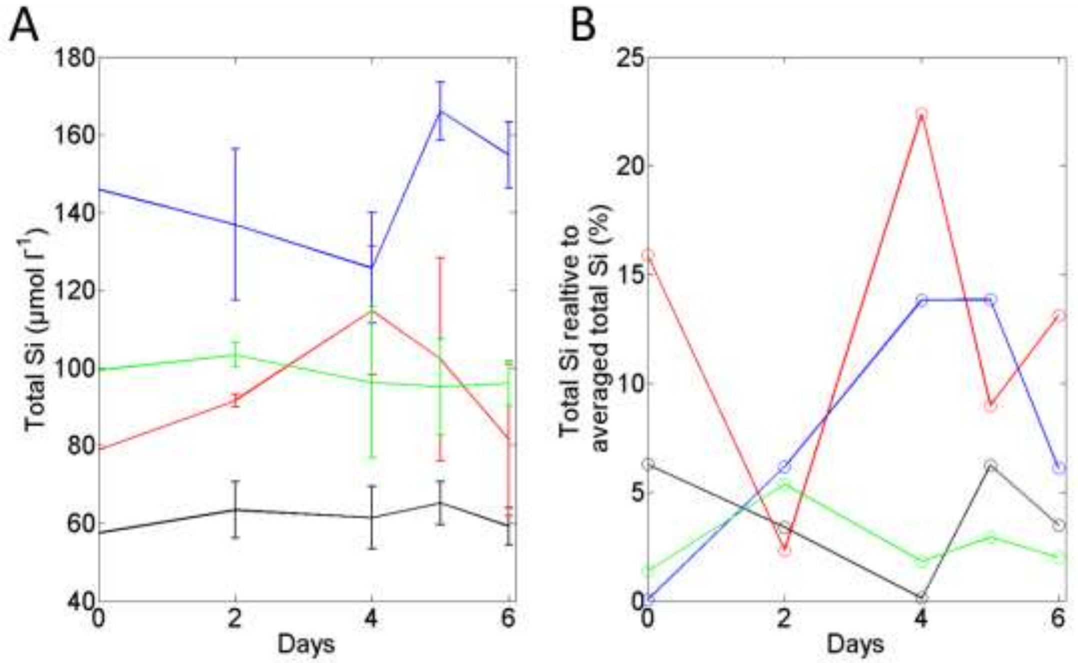

Figure S2: (A) Total Si (BSi + DSi). The black line represents 40m, the red line 60m, the blue line 110m and the green line 180m. Error bars are the propagated error from the standard deviations provided from the duplicates sample of both DSi and BSi. (B) Percentage of total Si (BSi + DSi) at the different sampling days over the averaged total Si over the entire duration of the experiment.

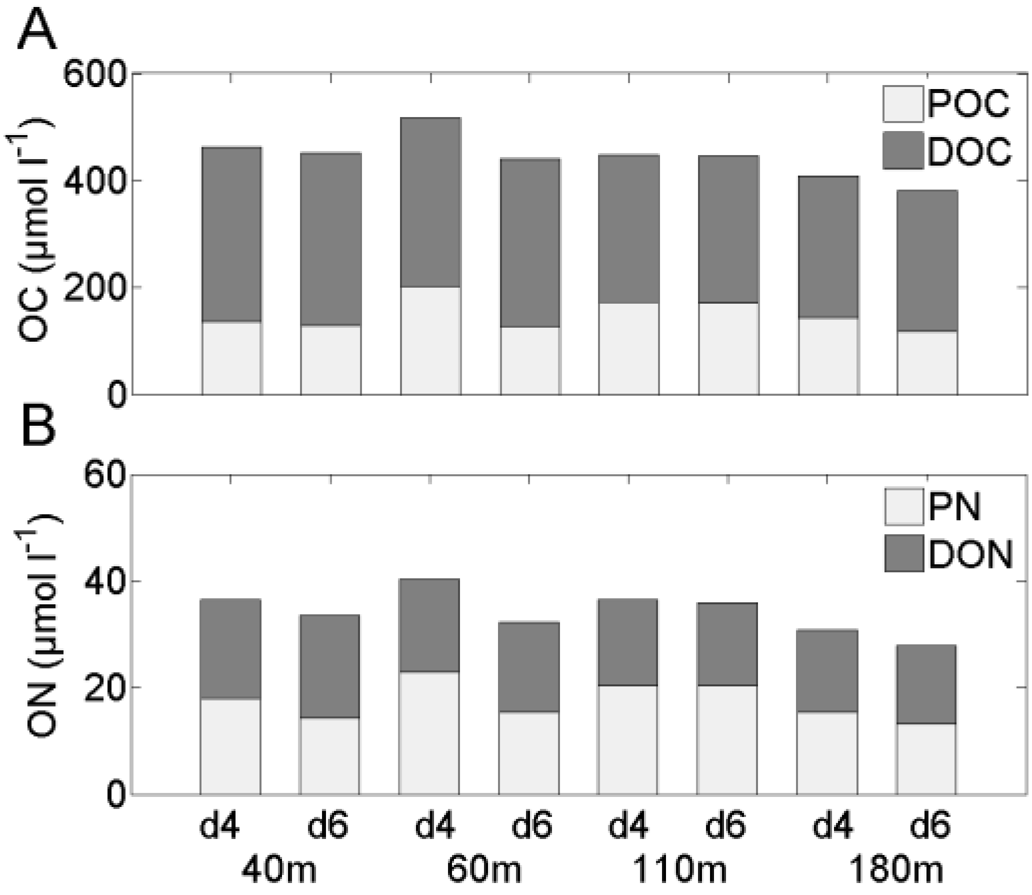

Figure S3: (A) Total organic carbon and (B) total organic nitrogen concentration (µmol l^{-1}) at day4 and day6 in the different depth treatments. The proportion of both DOC(DON) and POC(PN) is indicated in both panels

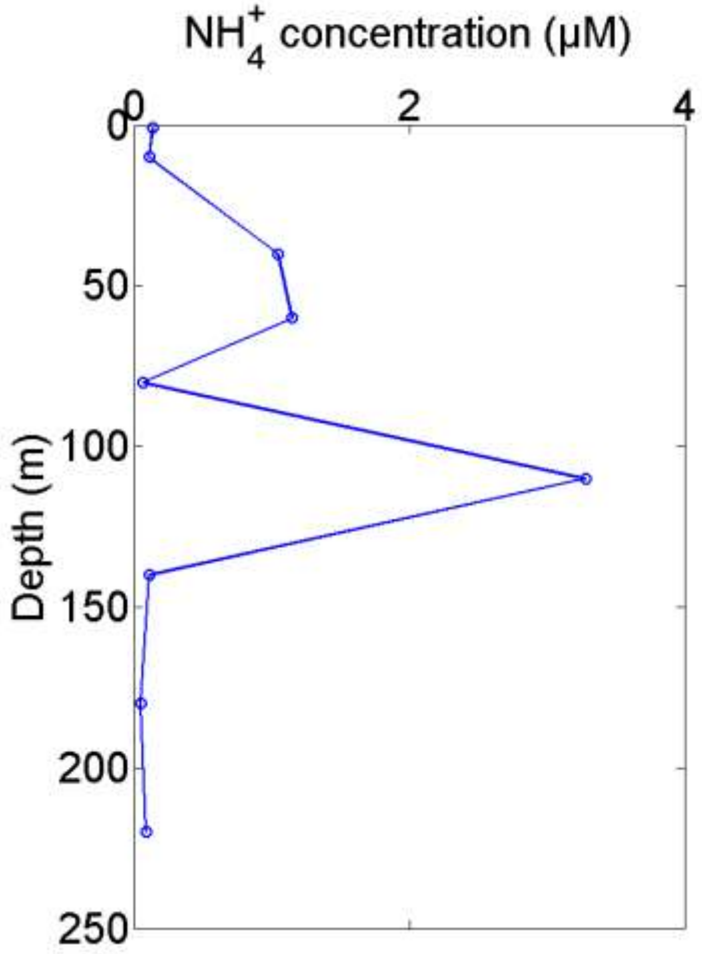

Figure S4: Vertical profile of ammonium concentration (μmol l^{-1}) at the sampling site.

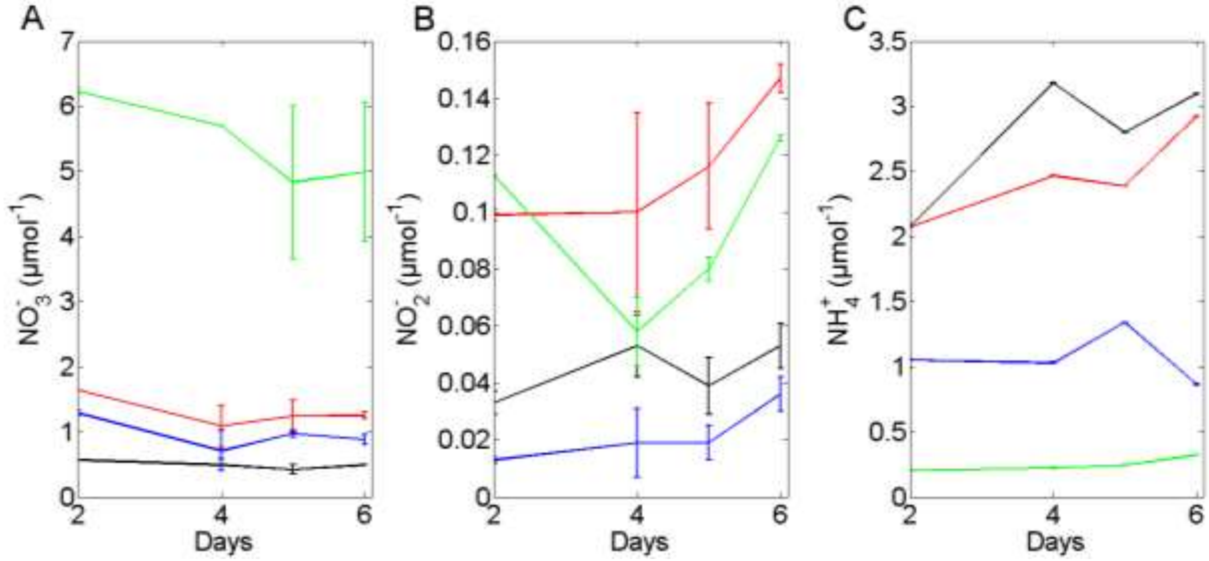

Figure S5: Nitrate, nitrite and ammonium concentrations (μmol l^{-1}) during our experiment. Error bars in panel A and B represent the standard deviation from replicates. Error bars on panel C represent analytical error, only one NH_4^+ sample was measured per treatment. Color code as in Figure S2.

Table S1: Concentration of total organic Carbon, Particulate Organic Carbon, Dissolved Organic Carbon in the different bottles during the experiment. Note that for d0, only POC concentration is given as DOC concentration was not measure in the trap material (see method section).

	40 m				60 m			
Concentrations in (µmol l^{-1})	d0	d2	d4	d6	d0	d2	d4	d6
Total OC	-	493	463	450 (43)	-	503	516	439 (64)
POC	149	121	136	129 (-8)	157	165	202	128 (37)
DOC	-	372	327	321	-	338	314	311
O_2 (mg l^{-1})	10.2	10.0	10.1	10.1	nan	11.5	11.5	12.0
	110 m				180 m			
Concentrations in (µmol l^{-1})	d0	d2	d4	d6	d0	d2	d4	d6
Total OC	-	518	447	446 (57)	-	481	408	380 (123)
POC	173	165	173	171 (-6)	168	138	143	118(20)
DOC	-	353	274	275	-	343	265	262
O_2 (mg l^{-1})	2.2	2.9	2.5	2.3	4.9	5.7	5.4	4.7

Table S2: Concentration of total Nitrogen, Organic Nitrogen, Particulate Nitrogen, Dissolved Organic Nitrogen, nitrate, nitrite and ammonium in the different bottles during the experiment. Note that for d0, only PN concentration is given as DON, nutrients and ammonium concentrations were not measure in the trap material (see method section). Losses of OC and ON in µmol l^{-1} calculated from the difference in OC(N) concentrations at day 6 relative to day 2 are indicated in brackets.

	40 m				60 m			
Concentrations in (µmol l^{-1})	d0	d2	d4	d6	d0	d2	d4	d6
Total N	-	41.47	40.10	37.03	-	45.63	43.95	36.57
Total ON	-	38.80	36.38	33.39 (5.4)	-	41.82	40.30	32.24 (9.6)
PN	19.01	15.75	17.89	14.18 (1.6)	19.98	21.98	22.79	15.32 (6.7)
DON	-	23.04	18.49	19.22	-	19.84	21.16	16.93
NO_3^-	-	0.57	0.49	0.49	-	1.64	1.09	1.25
NO_2^-	-	0.03	0.05	0.05	-	0.10	0.10	0.15
NH_4^+	-	2.07	3.2	3.10	-	2.07	2.47	2.92
DIN*		2.67	3.72	3.63		3.81	3.66	4.32
	110 m				180 m			
Concentrations in (µmol l^{-1})	d0	d2	d4	d6	d0	d2	d4	d6
Total N	-	43.98	38.13	37.78	-	45.70	39.12	33.14
Total ON	-	41.52	36.33 (5.6)	35.91	-	39.26	33.18	27.80(11.5)
PON	18.71	19.47	20.14(-0.8)	20.26	16.01	18.03	17.79	13.23(4.8)
DON	-	22.04	17.99	15.65	-	21.23	21.33	14.57
NO_3-	-	1.29	0.71	0.88	-	6.23	5.70	4.99
NO_2-	-	0.11	0.06	0.13	-	0.01	0.01	0.04
NH_4+	-	1.05	1.03	0.86	-	0.20	0.22	0.32
DIN*		2.46	1.80	1.87		6.45	5.94	5.34

*DIN is calculated as the sum of the concentrations of NO_3-, NO_2-and NH_4+

Table S3: Linear regression between POC:PN ratios, POC, PN and DOC concentration with time. The numbers in brackets are the standard error associated with the regressions

	All time steps	
	All depths	110m excluded
POC:PN	POC:PN = -0.0135(0.089)d + 8.465(0.361), r^2= 0.001, n = 20, p = 0.88	POC:PN = -0.0102(0.117)d + 8.289(0.471), r^2= 0.001, n = 15, p = 0.93
POC	POC = -2.605(2.4)d + 161.01(9.66), r^2= 0.061, n = 20, p = 0.29	POC = -3.991(2.450)d + 158.34(9.904), r^2= 0.104, n = 15, p = 0.12
PN	PN = -0.287(0.277)d + 161.01(9.66), r^2= 0.003, n = 20, p = 0.31	PN = -0.503(0.316)d + 19.236(1.273), r^2= 0.098, n = 15, p = 0.13
DOC*	DOC = -15.139(4.476)d + 373.5(20.143), r^2= 0.41, n = 16, p = 0.004	DOC = -13.443(5.322)d + 370.72(23.953), r^2= 0.32, n = 16, p = 0.030
	Day4, 5, 6 only	
	All depths	110m excluded
POC:PN	POC:PN = 0.046(0.266)d + 8.243(1.394), r^2= 0.003, n = 12, p = 0.86	POC:PN = 0.088(0.360)d + 7.980(1.828), r^2= 0.008, n = 9, p = 0.81
POC	POC = -13.651(8.99)d + 218.92(45.55), r^2= 0.187, n = 12, p = 0.16	POC = -17.823(8.12)d + 230.57(41.147), r^2= 0.323, n = 9, p = 0.05
PN	PN = -1.645(1.013)d + 26.068(5.135), r^2= 0.13, n = 12, p = 0.135	PN = -2.214(0.993)d + 27.971(5.032), r^2= 0.332, n = 9, p = 0.05
DOC*	DOC = -1.064(9.406)d + 300.01(47.656), r^2= 0.001, n = 12, p = 0.912	DOC = -1.6319(11.626)d + 309.04(58.902), r^2= 0.001, n = 9, p = 0.892

*DOC concentrations at day 0 are not considered because not measured.
